# Health-Related Quality of Life in Rugby Athletes: The Role of Dietary Supplements and Their Consumption

**DOI:** 10.3390/sports12100270

**Published:** 2024-10-08

**Authors:** Walter Sapuppo, Antonietta Monda, Davide Giacconi, Regina Gregori Grgič, Daniele Saccenti, Claudia Maria Mineo, Vincenzo Monda, Salvatore Allocca, Maria Casillo, Marcellino Monda, Girolamo Di Maio, Marco La Marra

**Affiliations:** 1 Department of Psychology, Sigmund Freud University Wien, 20143 Milan, Italy; w.sapuppo@milano-sfu.it (W.S.);; 2 Studi Cognitivi, Cognitive Psychotherapy School and Research Center, 20121 Milan, Italy; 3 Department for the Promotion of Human Science and Quality of Life, Telematic University San Raffaele, 00166 Rome, Italy; 4 Department of Economics, Law, Cybersecurity, and Sports Sciences, University of Naples “Parthenope”, 80133 Naples, Italy; 5 Department of Experimental Medicine, University of Campania “Luigi Vanvitelli”, 80138 Naples, Italy

**Keywords:** dietary supplements, health, quality of life, motivation, rugby, sports, athletes

## Abstract

This study investigates dietary supplement use among rugby players and their general health, focusing on prevalence and underlying motivations. Involving 92 athletes, it examines the relationship between supplement usage, motivations, and health outcomes using the 36-item Short Form Health Survey and a 24-item ad hoc questionnaire. Findings reveal a high frequency of supplement usage, motivated by desires to enhance performance, appearance, and mood. Significant differences in health-related quality of life are found between users and non-users, particularly in mental health, social functioning, and emotional stability. Motivations like performance enhancement and body shape manipulation were linked to altered health perceptions, indicating the psychosocial impacts of supplementation. This study emphasizes the need to consider the holistic effects of supplements on athlete well-being, advocating for a balanced approach prioritizing both physical and mental health. It calls for increased awareness among athletes, coaches, and sports professionals about the potential risks and benefits of supplement use and the importance of informed decision-making. Additionally, it highlights the need for further research to understand the mechanisms of supplement use and its impact on athlete health, aiming to enhance sports science and promote overall athlete well-being in competitive environments.

## 1. Introduction

Competition motivates athletes to strive for success in various pursuits, whether it involves defeating opponents or achieving personal goals, such as overcoming challenges or reaching specific sporting objectives [1,2,3,4,5,6,7]. To reach their goals, athletes can enhance their performances through various means, including physical conditioning, enhanced nutrition and sleep, stress mitigation, and mental readiness [8,9,10,11,12,13,14]. Furthermore, many pharmaceutical interventions have the potential to augment both strength and speed even further [15,16,17,18,19,20,21]. Among those substances, the most used by athletes at both amateur and professional levels are dietary supplements [22,23,24,25]. In fact, a survey conducted by Froiland et al. [26] found the usage rates of supplements among athletes to be as high as 89%. These dietary additives are usually employed to enhance athletic performance, speed up recuperation, and promote general health [27,28,29,30,31]. According to the International Olympic Committee [32], dietary supplements are defined as nutrients that are consumed in conjunction with the standard diet to enhance physical performance or provide health benefits [33,34]. They comprise a series of products meant to enhance athletic performance that contains vitamins, minerals, herbs, amino acids, dietary substances, concentrates, metabolites, extracts, or combinations of these ingredients that can be consumed only by oral intake [35,36,37]. Among dietary supplements, substances that can improve performance efficiency can be defined as ergogenic aids [38]. According to Froiland et al., [26], the most often consumed supplement was energy drinks, with a consumption rate of 73%. This was followed by calorie replacement products at 61.4%, multivitamins at 47.3%, and creatine at 37.2%. Elite athletes were found to utilize supplements more frequently than college or high school athletes, and women used supplements more often than males [26,39,40,41]. An examination of the rationales behind athletes’ utilization of dietary supplements reveals the following reasons for use: to enhance energy levels (61.2%), to facilitate fat burning and promote weight loss (38%), to supplement insufficient nutrition (35%), to enhance muscle growth (27.8%), and to alleviate tension and elevate mood (24.7%) [36,40,42]. However, the indiscriminate use of dietary supplements can lead to potentially both physical and mental risks, such as contamination, mood swings, cognitive changes, undesired drug interactions, and nutritional imbalances [43,44,45,46,47,48,49].

Among the multitudes of athletes practicing different kinds of sports and physical activities, the scientific literature is showing a growing interest in the assessment of rugby players. Rugby is a widely popular sport that boasts a global player base of over 8.5 million individuals. The sport’s professionalization in the 1990s has resulted in a notable rise in competition among players. Young rugby players now strive to attain specific physical attributes, such as greater muscular mass and strength, to meet the rigorous requirements of their sport [50,51,52,53,54]. This pertains to the notion of ‘larger is better’, as it is commonly considered that a rugby player with substantial physical mass exerts a more significant impact in the game in terms of strength, speed, and power [55,56,57,58,59,60]. For this reason, nutritional supplementation can be used to enhance performance and recovery indices or to modulate the shape of the body [61,62,63]. The pursuit of this goal can be enhanced through different kinds of supplements [64,65,66,67,68,69,70,71], including weight loss supplements, substances aimed at increasing muscle mass, or supplements designed to boost overall body strength [10,36,62,72,73,74].

Upon comparing the prevailing sports supplements utilized by professional rugby players in Europe [75,76,77,78], it was discovered that protein supplements were the most frequently used by rugby players from the European Super League [57]. This was followed by creatine (64%), while carbohydrate supplements were favored by 56% of the athletes. Caffeine was only utilized by 36% of rugby athletes as a sports supplement [57]. Rugby players primarily consumed whey protein and caffeine supplements, with 44% and 42% of players consuming each, respectively [79,80,81]. Although there is a need for more specialist literature on rugby, scientific interest in this sport is increasing. In fact, a growing branch of the literature is focusing on the use of dietary supplements to increase body mass and improve sports performance. However, there seems to be a limited understanding regarding the use of dietary supplements and their impact on rugby players’ mental health. Considering the high prevalence found in the literature of athletes who use dietary supplements, the aim of this study is to assess in a sample of rugby players the use of these substances and the reason underlying their consumption. Furthermore, differences in levels of health-related quality of life between athletes who reported using dietary supplements and those who did not will be analyzed.

## 2. Materials and Methods

### 2.1. Participants

Participants were recruited from the general population and through contact with rugby players and clubs on a voluntary basis. Subjects were recruited through online platforms (e.g., social media, e-mail), and the whole battery of questionnaires was administered online. In the following study, adult subjects (aged 18 years old or older) taking part in sport at a competitive or amateur level, or having done so in the past, were considered. From an initial sample of 174 athletes, 92 rugby players were identified and therefore included in the study. Only one subject was excluded due to a lack of full completion of the questionnaire.

### 2.2. Study Design and Procedure

This is a non-clinical cross-sectional study conducted between February and April 2023. Subjects who met the inclusion criteria were asked to give their consent to participate in the study by agreeing to informed consent regarding data processing for scientific and research purposes, which was presented to them before being able to access the questionnaires. Participants were informed about the purpose of the study and the anonymity of the data collection and analysis. This document, along with the questionnaires used and the methods of data collection and storage, was approved by the Ethics Committee of Sigmund Freud University, the Ethics Commission of the Faculty of Psychotherapy Science, and the Faculty of Psychology. The reference for this approval is GCP4Q7JFBO3P6I90070. To ensure confidentiality, any personal data that could make subjects recognizable were not collected.

### 2.3. Data Collection

A 24-item ad hoc questionnaire was created with the aim of investigating participants’ habits related to physical activity. In particular, the following information was collected: (1) the level of physical activity practiced by amateur athletes; (2) the type of sport practiced; (3) the frequency and duration of sports activity; (4) the type of dietary supplements; (5) the prevailing motivation for which they assume supplements; (6) the prevailing motivation behind practicing physical activity; (7) the agonistic level of physical activity; and (8) the level of physical activity practiced by professional athletes. Specifically, to collect information on supplement consumption and the motivations underlying their use, two direct multiple-choice questions were posed. To ensure that data on supplementation were linked to participants’ engagement in physical activity, they were asked to not disclose the potential use of supplements employed for the treatment of underlying health conditions. The questionnaire also included a brief collection of basic information such as gender, age, nationality, weight, and height to have a more precise description of the population surveyed.

To assess the quality of life of athletes, the Italian version of the 36-item Short Form Health Survey (SF-36; [82]) was utilized. The SF-36 is a self-report type of questionnaire, which aims to quantify health status and measure health-related quality of life. The questionnaire consists of 36 questions that can be divided into 8 subscales. Physical functioning (10 questions) assesses the influence of physical conditions on daily activities and the extent of physical exercise. Questions pertain to challenges encountered in doing routine tasks, such as ambulation or object manipulation. Limitations stemming from physical health (4 items) evaluate the extent to which physical health issues restrict work and social activities. Limitations arising from emotional issues (3 items) assess the influence of emotional factors on work performance and daily activities. Energy and dissatisfaction (4 items) gauge perceived energy levels and fatigue. Psychological well-being (5 items) examines mental health, encompassing anxiety and depression levels. Social engagement (2 items) measures the effects of physical and mental conditions on social relationships and activities. Pain (2 items) assesses the degree and frequency of physical pain and its impact on daily living and, lastly, general health perception (5 items) assesses the overall perception of health status and general well-being [82]. In this test, a low score is indicative of a low level of well-being and a high score indicates a high level of well-being. The global score was determined by performing an arithmetic mean of the subscales of the SF-36 questionnaire [83].

### 2.4. Data Analysis

First, descriptive statistics were computed on the data. Then, independent samples *t*-tests were used to compare the mean levels of health-related quality of life between subjects who consumed dietary supplements and those who did not. Additionally, multivariate analysis of variance (MANOVA) was used to test the effect of supplement consumption, competitive level, and gender on athletes’ health-related quality of life. One-way analysis of covariance (ANCOVA) was employed to conduct a comparison of the mean levels of subjects’ health-related quality of life, using the athletes’ self-reported motivations behind dietary supplement consumption as a grouping variable and their age as a covariate [84]. Age was selected as a covariate due to its significant association with health-related quality of life [85]. Normality was evaluated using the Shapiro–Wilk test and visual inspection of Q-Q plots. The statistical significance cut-off level was set at *p* < 0.05, 2-tailed. Data analysis was conducted utilizing statistical software, specifically R (Version 4.3.1) and RStudio for macOS (Version 2023.12.1).

## 3. Results

### 3.1. Descriptive Statistics

A total of 92 rugby players (83 males and 9 females) participated in the survey. The vast majority of them were Italian, except for two subjects (one with French and one with British nationality). The mean age of the sample was 33.01 (±13.52) years, ranging from 18 to 71 years of age. The mean body mass index (BMI) recorded within the sample was 28.08 (±4.17), ranging from a minimum of 20.06 to a maximum of 42.45. General health-related scores derived from SF-36 administration are outlined in Table 1.

In the remaining part of the assessment, participants reported having practiced regular physical activity for a mean of 18.74 (±10.17) years, ranging from a minimum of 4 to a maximum of 55 years. Subjects reported to have practiced rugby for a mean of 14.28 (±8.65) years, ranging from a minimum of 2 to a maximum of 43 years. Additional descriptive statistics relating to more detailed aspects of physical activity were calculated, including the regularity of physical activity, the prevailing motivation behind physical effort, and the competitive or professional level at which physical activity was practiced. Frequencies were broken down by gender and summarized in Table 2.

Turning to supplements and/or other substance usage, 57.6% of the participants reported that they consumed at least one supplement and/or other substance during their sporting practice, whereas the remaining 42.4% did not. The average number of supplements and/or other substances used by the sample was 1.73 (±1.89), ranging from a minimum of one to a maximum of seven dietary products currently taken. Among supplements and/or other substances consumers, self-reported motivations behind such behavior were as follows: to influence body appearance (3.2%), to influence athletic performance (34.8%), and to influence both body appearance and athletic performance (19.6%). Several types of supplements and other substances were used by the consumers subgroup, including whey proteins (40.2%), multivitamins or minerals (34.8%), creatine (27.2%), caffeine (16.3%), energy drinks (16.3%), carnitine (15.2%), analgesics (14.1%), cannabis (5.4%), and essential amino acids (4.3%).

### 3.2. Supplements Consumption and General Health

**Figure 1:**
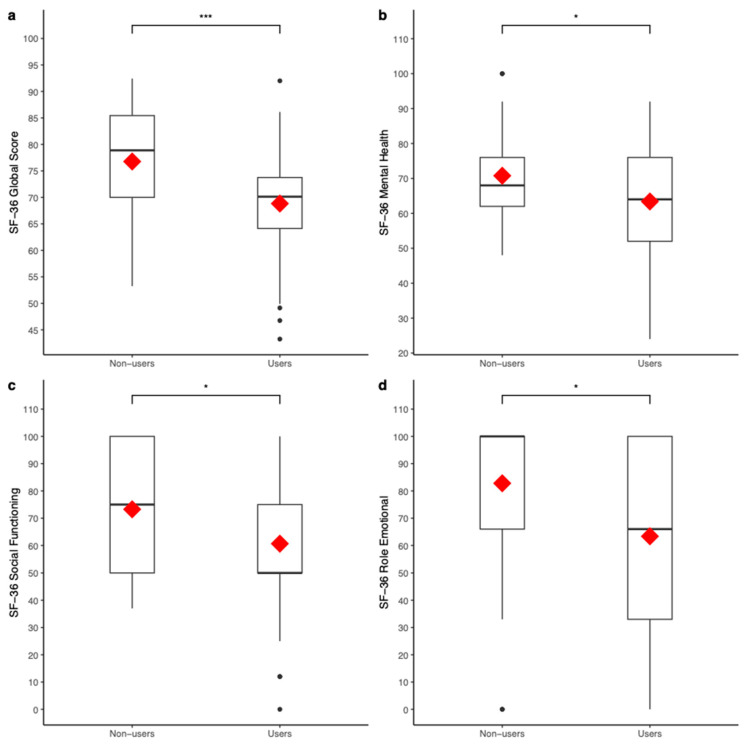
Box plots representing the mean (in red) of general health-related scores obtained by subjects who reported supplements and/or other substance consumption versus subjects who reported not consuming supplements and/or other substances. The SF-36 global score was compared between the two groups (**a**), as well as the following domains: mental health (**b**), social functioning (**c**), and role emotional (**d**). * = *p* < 0.05, *** = *p* < 0.001.

Independent samples *t*-tests were conducted to compare the general health-related mean scores obtained by the subjects who consume supplements and/or other substances with the ones who do not use them. A statistically significant difference in the SF-36 global scores was detected between the two groups (Group (Non-users): 76.77 ± 10.60; Group (Users): 68.83 ± 10.22; *t*(90) = 3.63, *p* < 0.001, *d* = 0.76; see Figure 1a). In particular, a statistically significant difference in the SF36 mental health (Group (Non-users): 70.77 ± 13.14; Group (Users): 63.40 ± 14.71; *t*(90) = 2.48, *p* < 0.05, *d* = 0.53; see Figure 1b), social functioning (Group (Non-users): 73.26 ± 23.26; Group (Users): 60.66 ± 25.28; *t*(90) = 2.44, *p* < 0.05, *d* = 0.52; see Figure 1c), and role emotional (Group (Non-users): 82.79 ± 30.58; Group (Users): 63.36 ± 40.47; *t*(90) = 2.52, *p* < 0.05, *d* = 0.54; see Figure 1d) sub-scales were obtained between the two groups. A statistically significant trend was also observed in the SF-36 vitality sub-scale while comparing the mean scores obtained by the two groups (*t*(90) = 1.97, *p* = 0.052, *d* = 0.42).

To further test the reliability of these results, we applied a MANOVA, using the global score of SF-36 and its eight subscales as dependent variables and the dichotomous variables supplement usage (i.e., Users versus Non-users), competitive level (i.e., Agonists versus Non-agonists), and gender as fixed factors. Overall, taken individually, supplements and/or other substance usage (Pillai’s trace = 0.10, *F*(9,72) = 0.84, *p* = 0.58), competitive level (Pillai’s trace = 0.18, *F*(9,72) = 1.77, *p* = 0.09), and gender (Pillai’s trace= 0.15, *F*(9,72) = 1.36, *p* = 0.22) did not significantly affect rugbyists’ general health-related scores, suggesting that there were not statistically significant differences between the groups with respect to all dependent variables examined simultaneously. However, tests of between-subjects effects highlighted that supplements and/or other substance usage, competitive level, and gender taken together significantly affect the global score of SF-36 (*F*(5,80) = 3.95, *p* < 0.01, *R*^2^ = 0.20) as well as its following domains: role emotional (*F*(5,80) = 3.35, *p* < 0.01, *R*^2^ = 0.17), social functioning (*F*(5,80) = 3.04, *p* < 0.05, *R*^2^ = 0.16), and mental health (*F*(5,80) = 2.78, *p* < 0.05, *R*^2^ = 0.15). A statistically significant trend was also observed when testing the effect of the three fixed factors on the SF-36 role physical sub-scale *(F*(5,80) = 2.29, *p* = 0.53, *R*^2^ = 0.13).

### 3.3. Motivations behind Supplements Consumption and General Health

**Figure 2:**
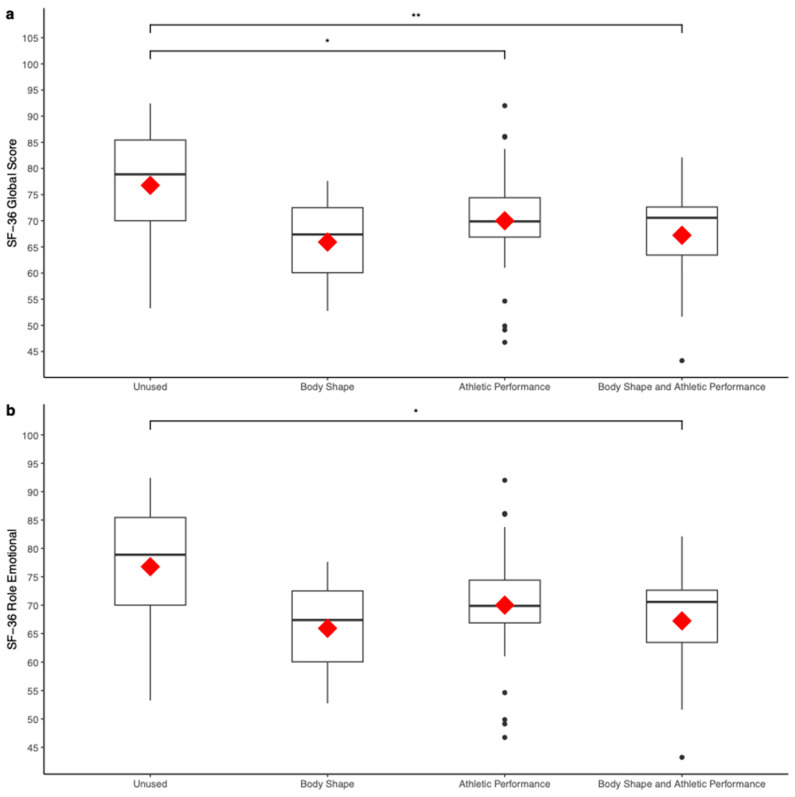
Box plots representing the mean (in red) of general health-related scores obtained by athletes according to their self-reported motivation behind supplements and/or other substance intake, controlling for age. The SF-36 global score was compared between the groups (**a**), as well as the role emotional domain (**b**). * = *p* < 0.05, ** = *p* < 0.01.

One-way ANCOVAs were conducted to compare the general health-related mean scores obtained by the subjects according to their self-reported motivations behind supplements and/or other substance intake, controlling for age. After adjusting for age, a statistically significant difference was detected in SF36 global scores between the groups (Group (Unused): 76.77 ± 10.60; Group (Body Shape): 65.92 ± 12.50; Group (Athletic Performance): 70.01 ± 10.42; Group (Body Shape and Athletic Performance): 67.22 ± 9.82; *F*(3,87) = 3.21, *p* < 0.05, *η*^2^ = 0.10; see Figure 2a). Pairwise comparisons of estimated marginal means highlighted a statistically significant difference between the scores obtained by subjects who do not use supplements and the scores of those who use supplements to affect athletic performance (*p* < 0.05, *d* = 0.64). Pairwise comparisons of estimated marginal means also highlighted a statistically significant difference between the scores obtained by subjects who do not use supplements and the scores of those who use supplements to affect body shape and athletic performance (*p* < 0.01, *d* = 0.93). A statistically significant difference was also detected in SF36 role emotional sub-scale between the groups (Group (Unused): 82.79 ± 30.57; Group (Body Shape): 44.33 ± 50.95; Group (Athletic Performance): 71.75 ± 38.95; Group (Body Shape and Athletic Performance): 51.61 ± 39.93; *F*(3,87) = 2.99, *p* < 0.05, *η*^2^ = 0.10; see Figure 2b). Pairwise comparisons of estimated marginal means highlighted a statistically significant difference between the mean scores obtained by subjects who do not use supplements and the scores of those who use supplements to affect body shape and athletic performance (*p* < 0.05, *d* = 0.87).

## 4. Discussion

This research investigates the use of dietary supplements among rugby players and their levels of general health, providing insights into the widespread utilization of supplements among athletes and the psychological motivation for their usage. Within the descriptive data, it was found that 53.3% of the participants indicated their engagement in physical activity to improve their physical and mental well-being. The outcome underscores a likely transition in individuals’ focus from performance-oriented to mental well-being-oriented. This level of consciousness is presumably of considerable significance in the domain of sports psychology, given that specific athletes experience mental and physical health consequences because of living a life centered on the quest for athletic prowess [86,87,88,89].

Focusing on dietary supplements, in line with the existing literature [17,36,57,90], a notable percentage of rugby athletes (i.e., 57.6%) disclosed utilizing supplements or other substances. Indeed, several investigations highlight the extensive utilization of these substances among athletes who aim to augment their athletic prowess, increase recuperation, or promote general health and wellness [13,15,80]. Another related aspect pertains to the reasons that drive rugby athletes to consume specific substances. A significant percentage of the sample (i.e., 34.8%) indicated that participants utilized supplements with the explicit intention of enhancing athletic performance. These data highlight the potential desire among athletes involved in this sample to enhance training efficiency and achieve a competitive edge [91,92,93]. Another group of participants (i.e., 19.6%) indicated instead that they utilized supplements to impact both their physical appearance and athletic performance, pointing to a possible comprehensive approach to supplementation that incorporates both visual appeal and performance-oriented objectives [11,58,81,94]. Given the acknowledgment and importance of mental health in the context of sports, there appears to be a prevailing notion that athletes should maintain optimal physical and mental fitness, while effectively managing various sport-related challenges, e.g., strict diet, rigorous competition, and declining performances [90,95,96,97,98]. In this framework, the use of dietary supplements could be related to the inner challenges that sport entails.

Multiple noteworthy insights are revealed by the results of independent group comparisons. In line with other studies [99,100,101], our analysis shows that athletes who reported the usage of nutritional supplements demonstrate lower average general health-related scores in comparison to those who did not consume them. Moreover, athletes who did not utilize dietary supplements consistently reported higher general health-related scores in terms of mental health, social functioning, and emotional regulation, indicating that these individuals had greater mental well-being, social integration, and emotional stability in comparison to those who did consume them. As underlined by recent research, athletes encounter considerable mental health challenges, encompassing depression, anxiety, fatigue, and body image issues [102]. Since challenges are exacerbated by the demands of performance, the necessity of physical fitness, and the need for rapid recovery from injuries, athletes may rely on supplements to manage these pressures, especially if they perceive that supplements can improve their performance, facilitate recovery, or boost their mental well-being [102,103]. In line with the contemporary literature, our results highlight the need for more research into the underlying relationship between supplementation and mental well-being to explore the possibility of a link between those variables [61,104,105]. One possible explanation for these results could be related to the inner characteristics of the sports discipline considered (i.e., the intermittent intensity of physical activity and the idea that it is necessary to be big and muscular to be a good player). Rugby, as a contact sport, necessitates substantial physical effort, resulting in significant energy expenditure during both matches and training sessions. In this scenario, the utilization of dietary supplements, both before and during the competitions, may yield favorable outcomes in terms of energy recovery and performance excellence [9,58,59,80]. However, it is important to note that excessive consumption of dietary supplements, such as creatine and caffeine, can potentially lead to adverse effects on both physical and mental well-being (i.e., nausea, excessive sleepiness, muscle pain [106,107,108,109]).

Another possible explanation regarding observed differences in health-related scores between supplement users and non-users concerns the significant pressure athletes encounter to succeed, prompting them to depend on supplements as an expedient solution. Rugby players, who must appear strong and fit, may feel pressure to match body image norms [52]. If the expected results are not obtained, this focus on body image can lead to negative mental health effects such as body dysmorphia, low self-esteem, and dissatisfaction with their looks, which can, in turn, promote depression and anxiety, hence reducing well-being [52]. Over time, dependence on supplements may undermine an athlete’s faith in their innate skills, leading to mental health issues such as anxiety, stress, or feelings of inadequacy when their performance fails to meet expectations [110,111]. Athletes with anxiety or depression might hope supplements enhance their mental or physical health [38]. In this situation, supplements may reflect their poor health rather than cause lower mental health scores [110,112]

Besides supplement intake, we observed that health-related quality of life is influenced by gender and competitive sport level. The limited representation of women in the sample may result in biases or underrepresentation in the data, complicating the generalization of findings across genders. Nevertheless, results appear consistent with the existing literature on the topic, which highlights the presence of gender differences in athletes’ mental health [113], as well as significant differences in their engagement dimensions (e.g., confidence, vigor, dedication, and enthusiasm) based on the interplay of gender, age, and competitive level [114]. For this reason, exploring the potential gender differences in supplement usage or motivation may represent an important means for future research projects.

We also noticed substantial relationships between dietary supplement usage motivations and general health-related outcomes, suggesting that rugby players with varying reasons behind supplement intake have distinct perceptions of their health. More specifically, athletes who indicated the utilization of supplements to manipulate body shape or enhance athletic performance showed lower health-related quality of life in comparison to those who abstained from supplement usage. This highlights the possible psychosocial ramifications of supplement consumption [115]. A particular perspective is that athletes facing health issues may be more inclined to utilize supplements to enhance or control their condition. In athletes, diminished health-related scores might be associated with overtraining or insufficient recovery [116], perhaps leading them to depend on supplements for compensation. Athletes who overtrain may encounter chronic weariness, injuries, or compromised immune systems, prompting them to use supplements as a temporary solution instead of modifying training intensity or ensuring adequate rest [117]. Athletes with chronic or recurrent health problems may endure psychological stress or anxiety regarding their performance or general well-being, leading them to seek supplements [118]. Likewise, rugby players who utilize supplements to enhance body shape and athletic performance demonstrated significantly lower emotional regulation capacities in comparison to those who do not use such supplements. The results provide an overview indicating that incentives to consume supplements, which are linked to enhanced body image and performance, might affect emotional well-being and role functioning [94,119,120,121,122,123,124,125].

The current study is, however, subject to several limitations. The cross-sectional approach employed in this work precludes the establishment of cause–effect relationships, underscoring the need to conduct longitudinal investigations to clarify temporal relationships. In fact, the relationship between dietary supplements and athletes’ health-related quality of life might be more intricate, with other variables that could influence this association, such as food, exercise intensity, sleep quality, and mental health, significantly influencing athletes’ general health [126]. Additionally, athletes with existing health issues, both physical and psychological, may be more predisposed to utilizing supplements to alleviate their conditions; conversely, excessive or inappropriate usage of supplements may lead to adverse health effects over time, including nutrient intake imbalances or psychological stress due to dependency on supplements [126,127]. Despite the powerful insight given by the literature on the potential influence of participants’ physical condition on the use of dietary supplements, the aim of the current study specifically asked participants not to disclose supplements used to treat medical conditions.

Another limitation of this study is that it was limited to rugby players; thus, the generalizability of these findings to other sports is still being determined. Furthermore, due to the preponderance of male athletes over female athletes, the sample’s characteristics precluded us from identifying gender differences. Moreover, considering the small sample size and the fact that participation in this study was voluntary and handled entirely online, it is possible that the characteristics of participants were non-homogeneously distributed among the sample, hence creating a selection bias, which could have influenced the generalizability towards all rugby players. To overcome these constraints and provide a more thorough understanding of the psychological and physical characteristics of rugby players, future studies should employ data-driven statistical techniques or expand the sample size and heterogeneity in terms of sports disciplines. Future research should focus on conducting a longitudinal study to examine the potential impacts and long-term intake of dietary supplements among rugby athletes. In particular, it would be interesting to assess, among athletes, the quantities and impact on general health-related outcomes of these non-medical and non-regulated substances. Moreover, future research could focus on investigating whether the use of nutritional supplements and the reason underlying supplementation have an impact on specific cognitive characteristics such as cognitive fusion, cognitive flexibility, and coping strategies [128,129,130,131,132,133]. These contributions can give a possible direction about the understanding of how dietary supplements can affect athletes’ general health.

## 5. Conclusions

This study explores the complex relationship between dietary supplement consumption and health outcomes in rugby players, highlighting both possible advantages and risks. The varied motivations behind supplement use (e.g., performance enhancement versus aesthetic improvement) were discussed, enriching the knowledge of how these factors may influence athletes’ health-related quality of life and providing a possible framework for the psychological determinants of supplement intake. The findings indicate that whereas supplements are frequently utilized to improve performance, accelerate recovery, and sustain well-being, their consumption may also result in unforeseen physical and psychological effects. Consequently, the necessity of embracing a balanced, informed strategy toward supplementing is highlighted throughout this study. Athletes and coaches must possess an extensive understanding of the interactions between supplements and both physical and mental wellness. They should avoid dependence on supplements as immediate solutions and instead evaluate their comprehensive lifestyle, encompassing nutrition, training, and mental health management. Supplementation programs must be customized to the specific requirements of athletes, considering their health status, performance objectives, and mental health concerns. This may avoid the overutilization or improper usage of supplements and reduce possible associated dangers. Indeed, it would be beneficial to evaluate the impact of psychoeducational programs on the after-effects of supplement consumption to promote a healthy approach to their use. Sports experts, such as nutritionists, psychologists, and medical personnel, should collaborate to ensure that athletes’ supplementation habits are consistent with their overall health objectives. This interdisciplinary strategy can assist athletes in preserving both physical and psychological well-being. Additionally, further research is required to better understand the mechanisms underlying supplement use and its effect on the health outcomes of athletes. This could bring additional insight into the field of sports psychology and improve the overall well-being of athletes participating in competitive settings.

## Figures and Tables

**Table 1:** Summary of general health-related characteristics.

Variable	Mean ± Standard Deviation	Minimum	Maximum
Global Score	72.20 ± 11.05	43.25	92.43
Physical Functioning	94.13 ± 11.18	40.00	100.00
Role Physical	79.35 ± 30.0	0.00	100.00
Role Emotional	71.60 ± 37.68	0.00	100.00
Social Functioning	66.00 ± 25.11	0.00	100.00
Bodily Pain	77.62 ± 18.41	32.00	100.00
Vitality	56.74 ± 12.76	25.00	85.00
General Health	64.01 ± 13.06	40.00	100.00
Mental Health	66.52 ± 14.46	24.00	100.00

**Table 2:** Summary of physical activity-related specifications.

Variable	Males	Females	Total
Physical activity regularity			
			
My physical activity has been generally constant over the years	63 (75.9%)	9 (100%)	72 (78.3%)
I used to practice more physical activity than I do now	18 (21.7%)	0 (0.0%)	18 (19.5%)
I used to practice less physical activity than I do now	2 (2.4%)	0 (0.0%)	2 (2.2%)
Prevailing motivation behind practicing physical activity			
			
Competition	16 (19.3%)	0 (0.0%)	16 (17.4%)
Entertainment and social occasions	8 (9.6%)	3 (33.3%)	11 (12.0%)
Improvement of body image	2 (2.4%)	0 (0.0%)	2 (2.2%)
Improvement of physical and psychological health	44 (53.0%)	4 (44.5%)	48 (52.1%)
Improvement of athletic performance	13 (15.7%)	2 (22.2%)	15 (16.3%)
Agonistic level physical activity			
			
I currently compete at the agonistic level	68 (81.9%)	9.00 (100%)	77 (83.7%)
Not at the moment, but I have competed at the agonistic level in the past	15 (18.1%)	0.00 (0.0%)	15 (16.3%)
Professional-level physical activity			
			
I’ve never competed at the professional level	81 (97.6%)	9 (100%)	90 (97.8%)
Not currently, but in the past I competed at the professional level	2 (2.4%)	0 (0.0%)	2 (2.2%)

## Data Availability

Collected data are available by contacting the corresponding author under proper request.
